# Supplementary figures and images for: An integrative taxonomy approach reveals Saccharomyces chiloensis sp. nov. as a newly discovered species from Coastal Patagonia
Source: PLoS Genet. 2024 Sep 6;20(9):e1011396. doi: 10.1371/journal.pgen.1011396 (PMC11410238; doi:10.1371/journal.pgen.1011396)

A

*RIP1*

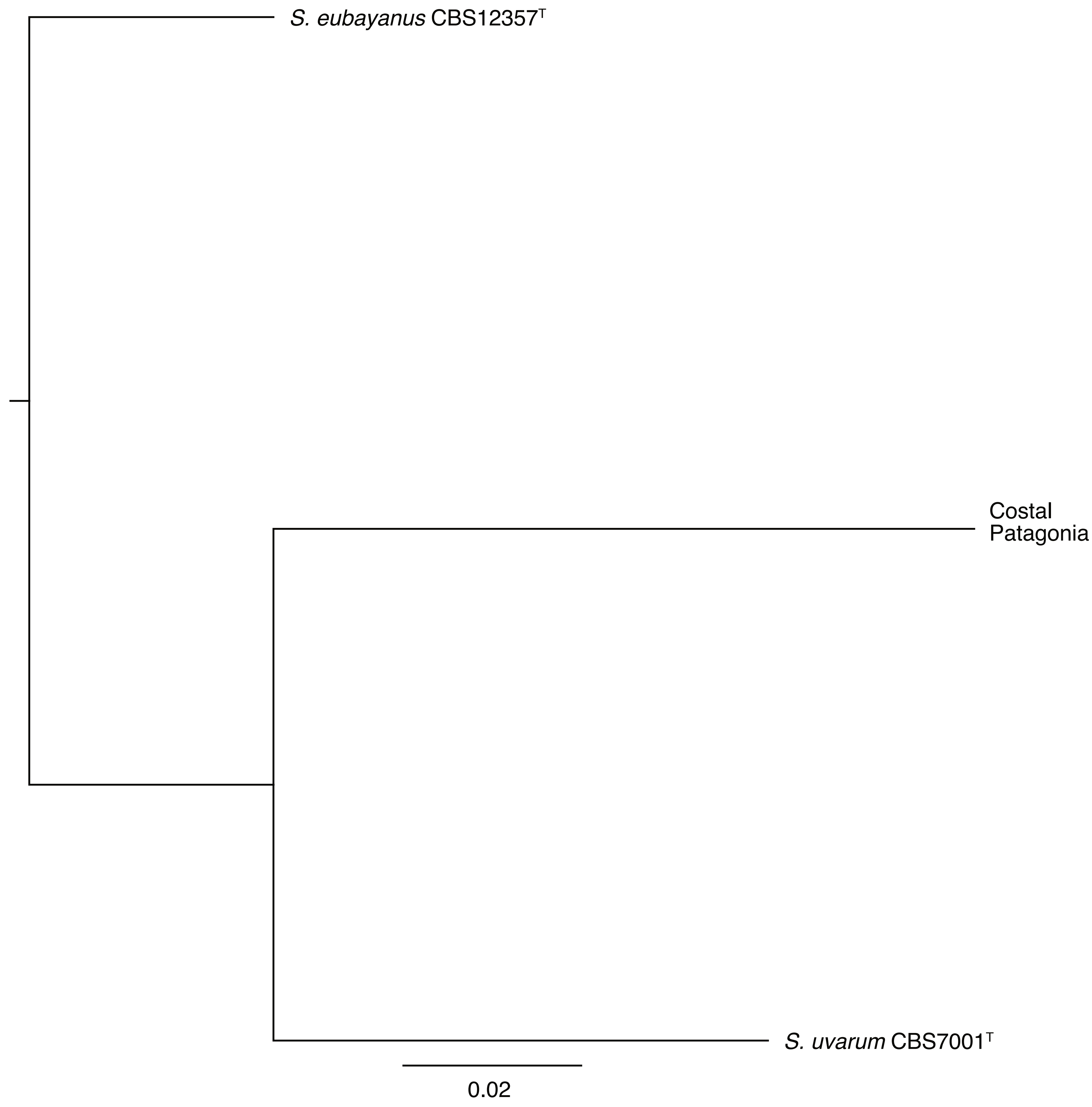

B

26S rRNA *D1/D2* and *ITS*

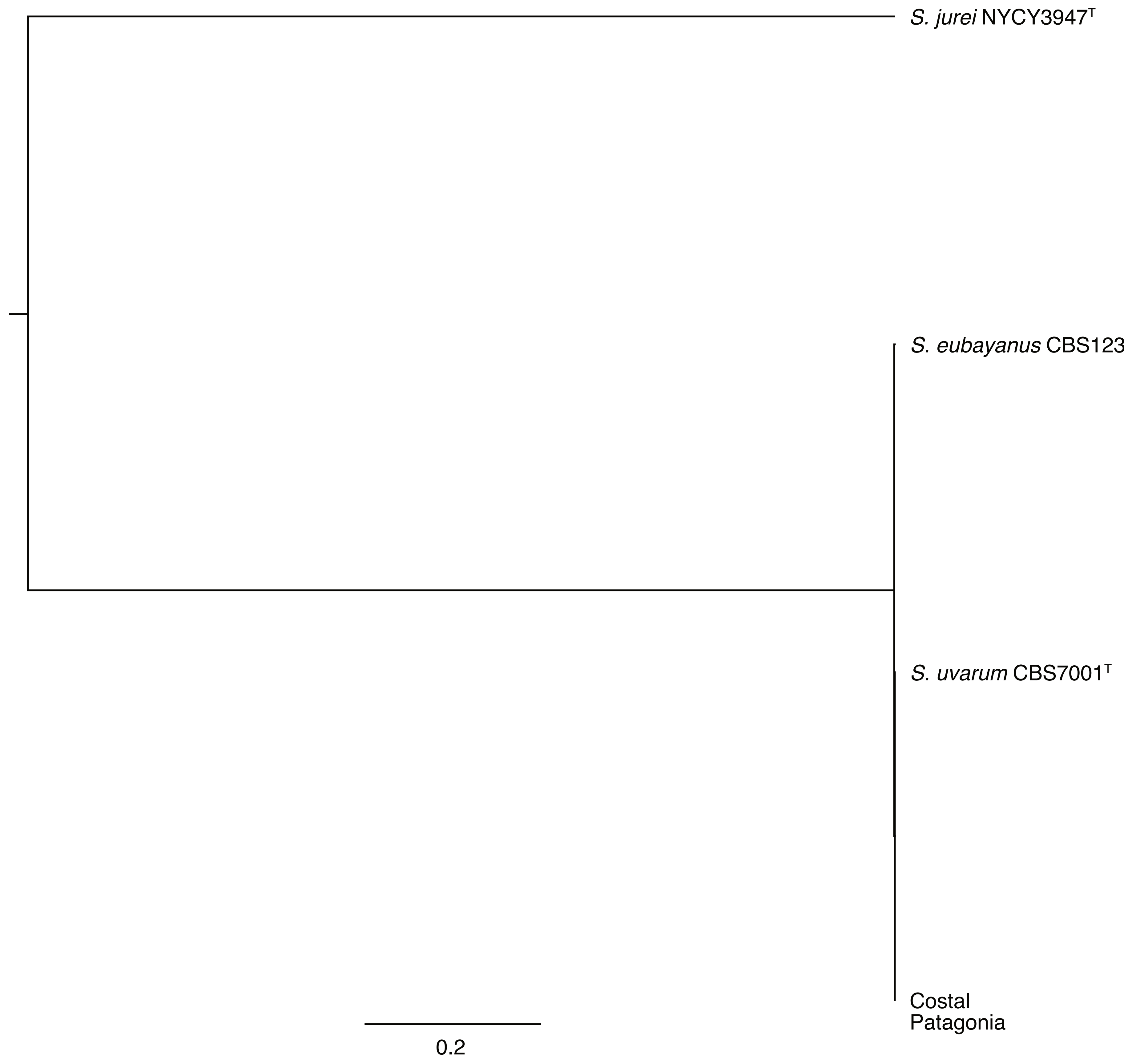

C

26S rRNA *D1/D2* and *ITS*

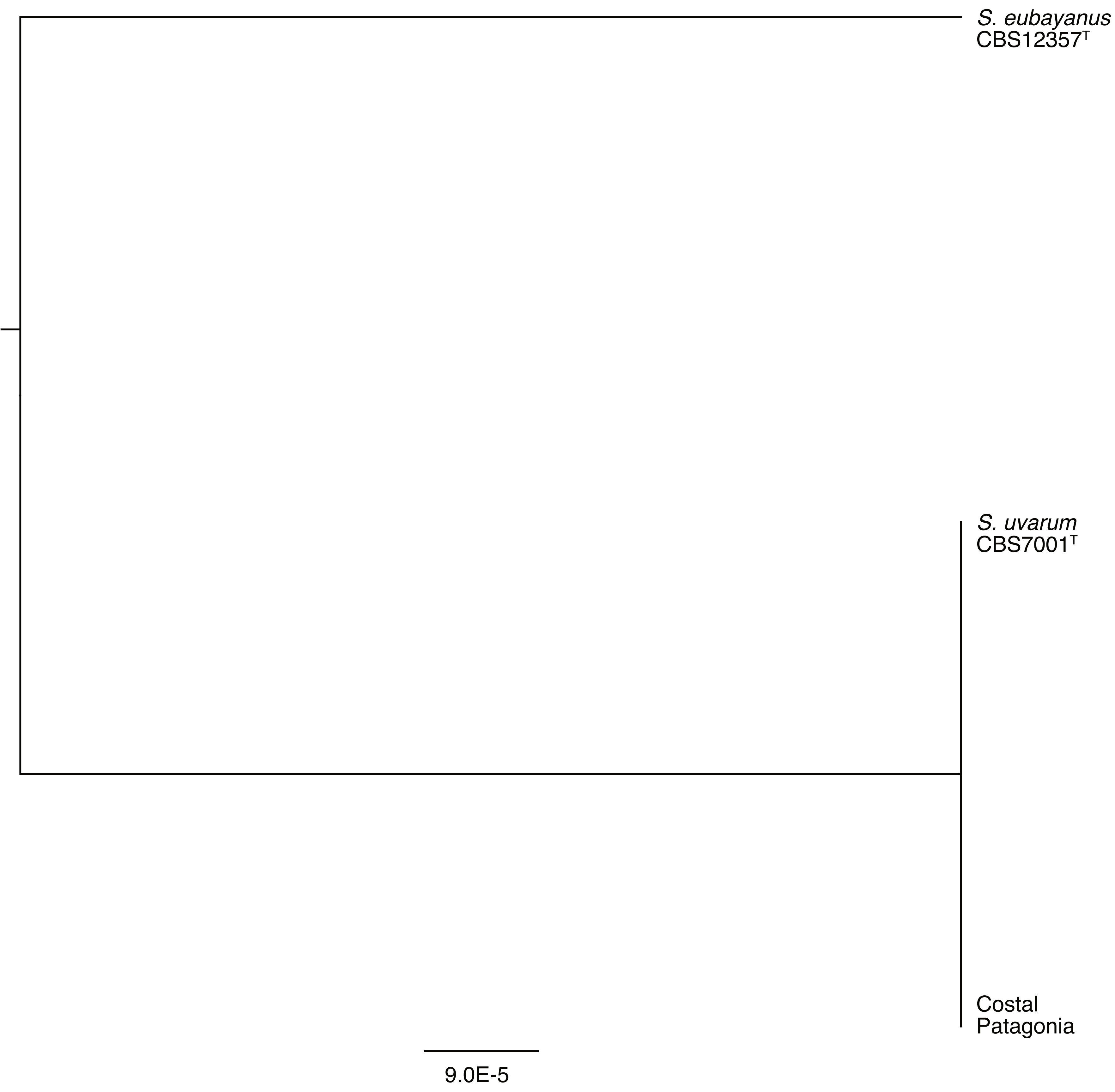

Supplementary Figure 1

Supplement: S1 Fig — (A) RIP1 NJ phylogram. The tree was built based on the sequences of the RIP1 marker. The alignment was performed with MUSCLE. The distance metric is the number of substitutions. Bar, Substitutions per site. Bootstrap values are shown for each node. (B) Concatenated LSU-ITS-SSU NJ phylogram. The alignment was performed with MUSCLE. The distance metric is the number of substitutions. Bar, Substitutions per site. S. jurei sequences as outgroup. (C) Same as B but S. eubayanus as an outgroup. (PDF) [file pgen.1011396.s001.pdf]

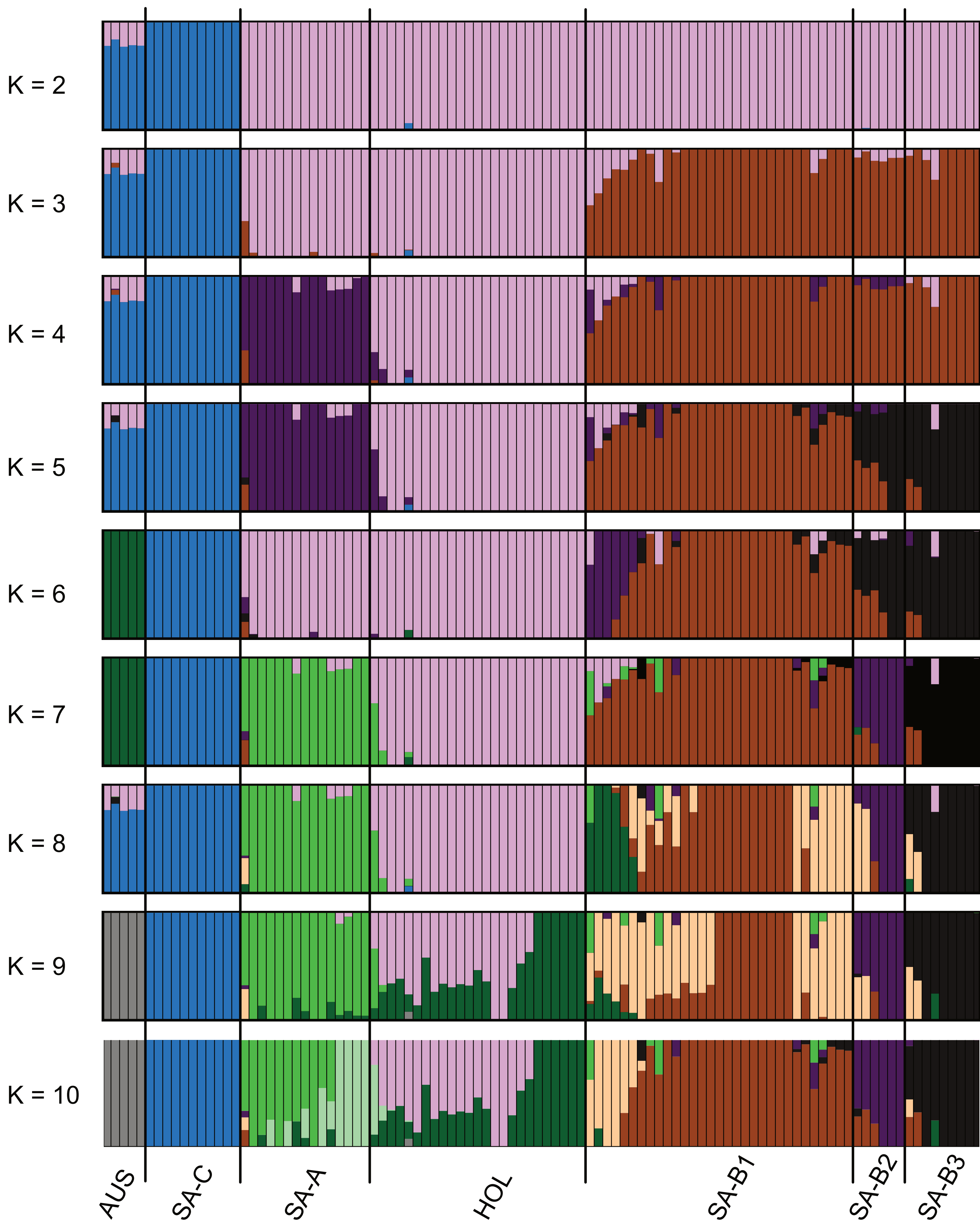

Supplementary Figure 2

Supplement: S2 Fig — Admixture plots (k = 2 to k = 10) for 100 individuals. Each color depicts a different lineage. (PDF) [file pgen.1011396.s002.pdf]

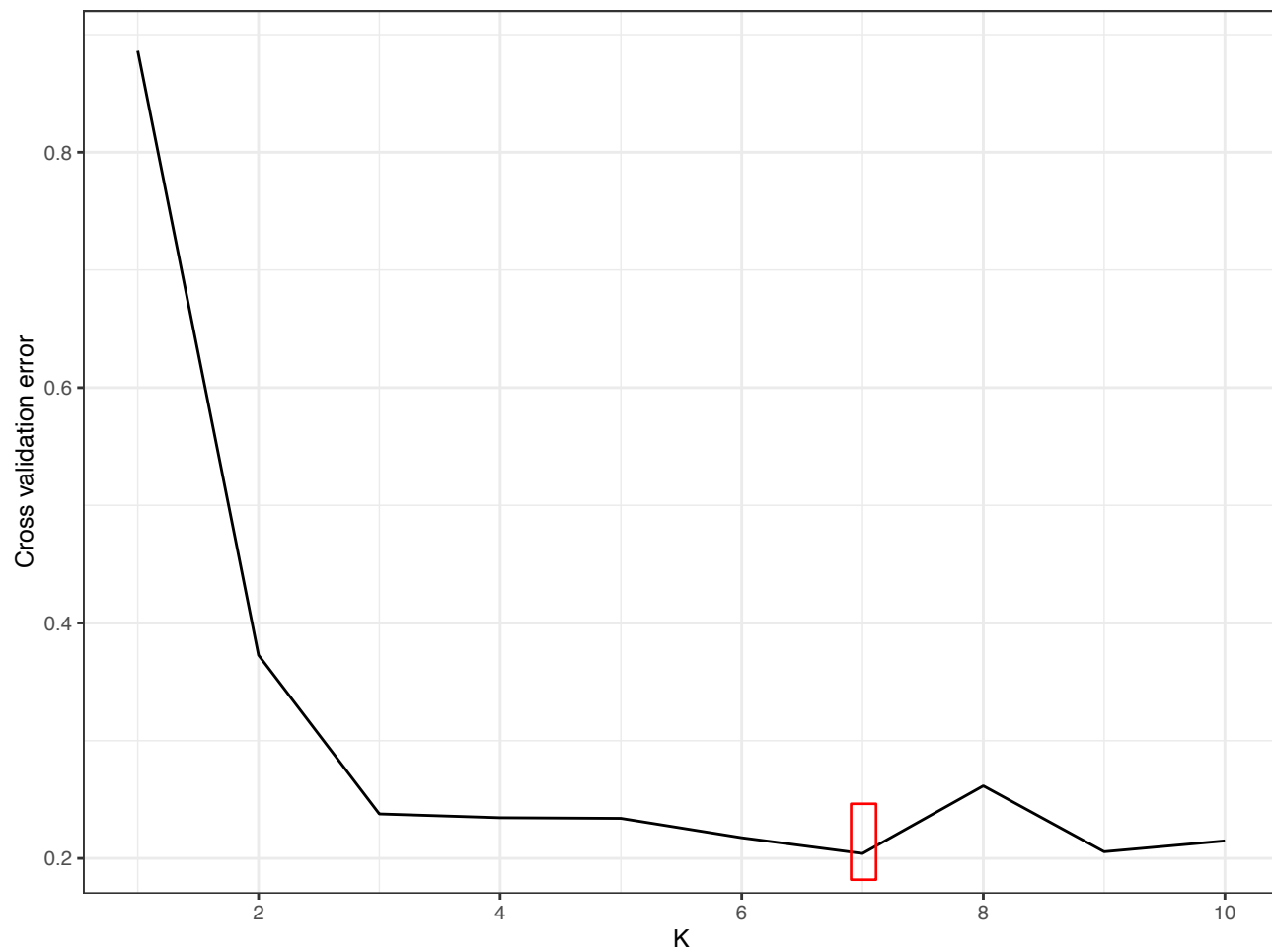

Supplementary Figure 3

Supplement: S3 Fig — For each k (1–10) the cross-validation error was estimated. A red square was shown to indicate the lowest value. (PDF) [file pgen.1011396.s003.pdf]

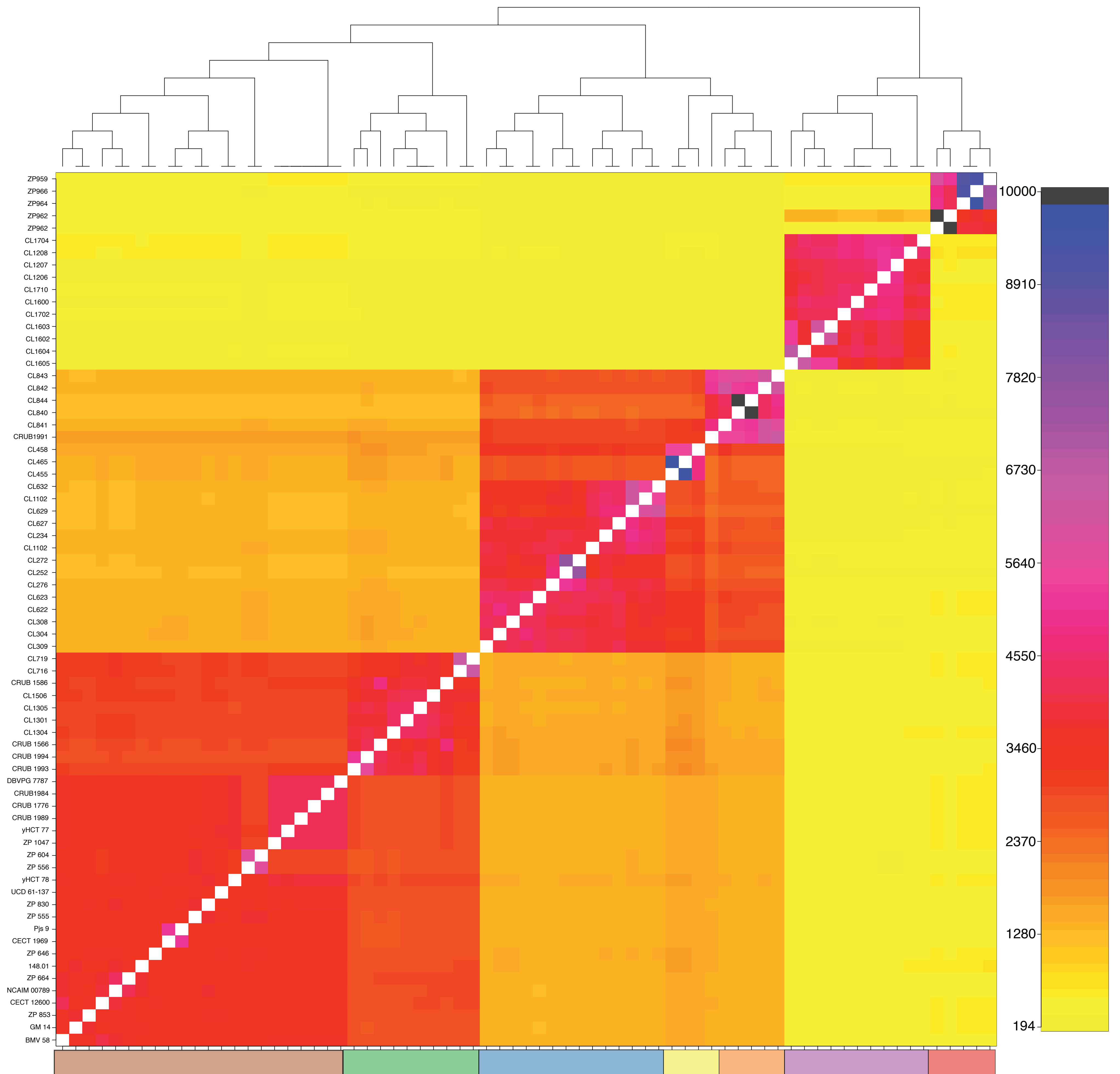

Supplementary Figure 4

Supplement: S4 Fig — The heatmap was obtained using fineSTRUCTURE chunk counts. Each row and column represent a strain, and the color scale indicates genetic sharing (yellow = low sharing, blue = high sharing). The tree shows the clusters inferred from the co-ancestry matrix. Populations and subpopulations can be inferred from the presence of darker colors in the diagonal. The strain matrix is orderly correlated. Below the figure colors represent lineages (brown: Hol, green: SA-A, blue: SA-B1, yellow: SA-B2, orange: SA-B3, Purple: SA-C and red: AUS). (PDF) [file pgen.1011396.s004.pdf]

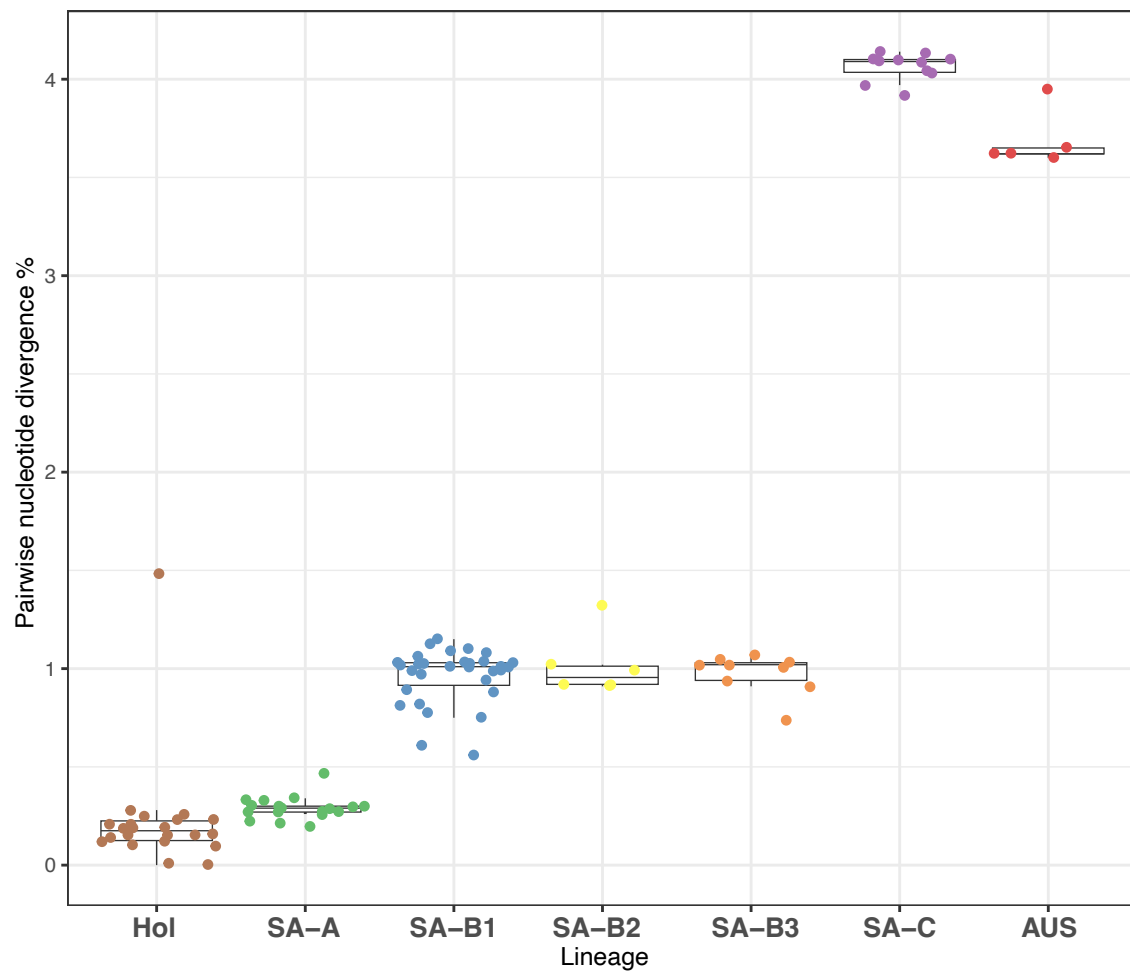

Supplementary Figure 5

Supplement: S5 Fig — The nucleotide divergence relative to the S. uvarum CBS7001T strain is shown. Lineages and colors are depicted as follows: South America (SA-A (green), SA-B1 (blue), SA-B2 (yellow), SA-B3 (orange) and SA-C (purple)), Australia (AUS, red) and Holarctic (HOL, brown). (PDF) [file pgen.1011396.s005.pdf]

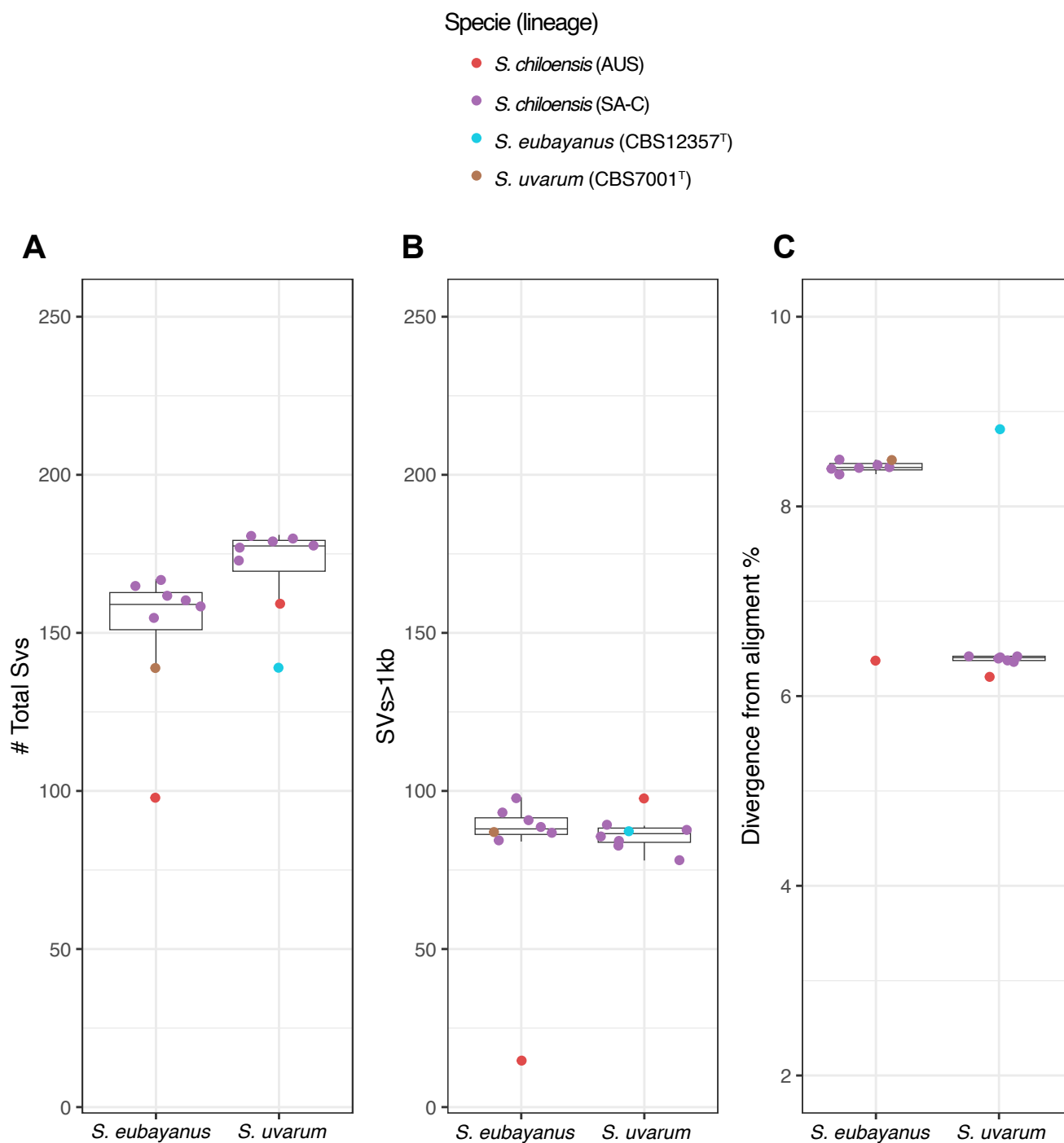

Supplementary Figure 6

Supplement: S6 Fig — (A) Total number of Structural variants (SVs), (B) SVs larger than 1 kb, and (C) Divergence from alignment. Colors depict S. chiloensis sp. nov (AUS, red), S. chiloensis sp. nov (SA-C, purple), S. eubayanus (light blue) and S. uvarum (brown). (PDF) [file pgen.1011396.s006.pdf]

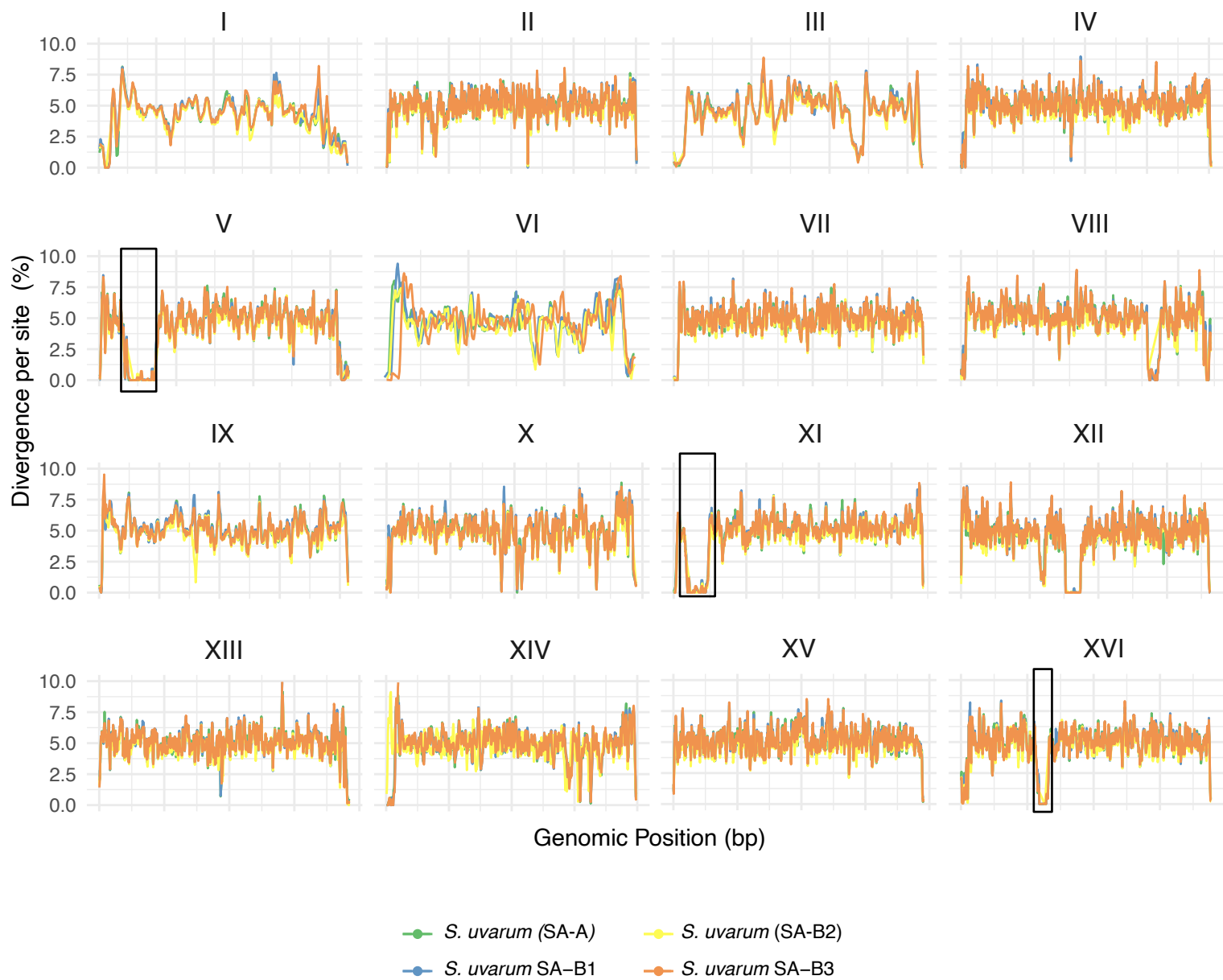

Supplementary Figure 7

Supplement: S7 Fig — Each plot represents the % divergence per site relative to S. chiloensis reference strain CBS18620T on a 1 kb window (x-axis values are in bp). S. uvarum lineages are color-coded according to the key. (PDF) [file pgen.1011396.s007.pdf]
